# The “hot potato” topic: challenges and facilitators to promoting respectful maternal care within a broader health intervention in Tanzania

**DOI:** 10.1186/s12978-018-0589-1

**Published:** 2018-09-12

**Authors:** Shannon A. McMahon, Rose John Mnzava, Gaudiosa Tibaijuka, Sheena Currie

**Affiliations:** 1 Heidelberg Institute of Global Health, Medical Faculty and University Hospital, University of Heidelberg, Heidelberg, Germany; 2 Department of International Health, Social and Behavioral Interventions Program, Johns Hopkins Bloomberg School of Public Health, Baltimore, USA; 3 Jhpiego/Tanzania, an affiliate of Johns Hopkins University, PO Box 9170, Dar es Salaam, Tanzania; 4 Jhpiego/USA, an affiliate of Johns Hopkins University, 1615 Thames St., Baltimore, 21231 MD USA

**Keywords:** Respectful maternity care, Abuse, Disrespectful care, Maternal health, Gender, Programming, Process evaluation, Program learning

## Abstract

In recent years, mistreatment during childbirth has captured the public health and maternal health consciousness as not only an affront to women’s rights but also a formidable deterrent to the uptake of facility-based childbirth - and thus to reductions in maternal mortality. The challenge ahead is to determine what can be done to address this public health problem. A modest but growing body of research has demonstrated that interventions to foster Respectful Maternity Care (RMC) can enact change, albeit in the relatively controlled context of a trial or study. Herein we describe our experiences in weaving elements of RMC across tiers of an existing maternal and newborn health program. As a commentary, this document does not outline program results, but instead highlights challenges and facilitators to promoting RMC within a large-scale, multi-district health platform. We conclude with lessons learned during the process and urge that others share their program learning experiences in an effort to strengthen the knowledge base on what works and what does not work in terms of addressing this complex, context-sensitive issue.

## Plain text summary

While the academic literature continues to expand in terms of defining the nature and scope of abusive care or mistreatment toward women during childbirth, there is less consensus and documentation about what can be done to address this problem. This is in part because addressing abuse and promoting respect is hard and needs to be addressed at all levels of the health system; there is no discrete, technical fix that can shift individual attitudes, transform patient-provider relationships and/or upend deep-seated social norms. Despite these challenges, addressing abuse and promoting respect is important - particularly in terms of getting more women to deliver their babies in health facilities and ensuring that care is acceptable. This commentary joins a growing conversation regarding how the promotion of respectful care can be done. The commentary does not emphasize results of an intervention, but rather talks about the challenges and breakthroughs experienced while trying to get an intervention underway. We present this as a means to support programmers in similar settings as they embark on interventions to mitigate disrespect.

## Background

Since 2010, a flurry of research has been published regarding the existence of disrespect and abuse (D & A) toward women during childbirth [1–3]. A topic that once scarcely attracted attention in global health spheres has now become a battle cry among programmers, academics, donors and other stakeholders in the fields of maternal health, public health and human rights [4]. So elevated is the topic in the broader global health discourse, that the World Health Organization’s (WHO) 2015 Quality of Care framework lists the provision of respectful, dignified care as a tenet of quality – on par with more traditional quality domains such as provider competence and health system infrastructure [5].

While the need to promote respectful, dignified care is now widely viewed as an essential component to improve careseeking [5], questions remain in terms of how to best address this complex, context-sensitive issue. Recent research has highlighted the drivers of D & A, emphasizing how factors operating at the societal level (gender disparities, power imbalances, inadequate understanding of human rights) and at the health system level (inadequate equipment, staffing, training, compensation and supervision) often foment disrespect [1, 6]. Findings from at least three recent interventions have been published, highlighting outcomes of implementing programs that aim to promote respectful maternity care (RMC). Abuya and colleagues (2015) state that their intervention in 13 facilities in Kenya led to a reduction in most forms of D & A [7]. Ratcliffe and colleagues (2016) note that their intervention in one facility in Tanzania fostered improved patient-provider relations [8]. Neither study included a comparison group, and thus could not control for secular trends. Most recently, the “Staha” program in one intervention (and one control) facility in Tanzania reported a 66% reduction in the odds of a woman experiencing D & A after the implementation of a community and health system intervention [9]. To date, we note that less emphasis has been placed on the challenges and facilitators to implementing RMC interventions, and we are unaware of literature that describes how to integrate RMC components into a broader maternal health platform.

In 2014, Jhpiego Tanzania decided to weave components of RMC into its marquee Maternal and Child Survival Program (MCSP). MCSP is a USD $32 million program undertaken across two regions of Tanzania with a catchment area of approximately two million people in Mara and three million people in Kagera. The decision to incorporate RMC into MCSP stemmed from recognition – at a global level and within country, on behalf of both Jhpiego and MCSP’s donor – of a burgeoning body of evidence on the importance of RMC in promoting facility-based childbirth. The implementing team initially viewed RMC programming as more or less in sync with other maternal health interventions, and intended to undertake a program in a manner similar to the way other reproductive, maternal, newborn, child and adolescent health programs in Tanzania are implemented; a process that generally involves agreeing on an evidence-based intervention for a given health issue, sharing insights on the given health issue and its attendant proposed intervention with the Ministry, gaining Ministry consensus and approval to implement the intervention, and ultimately proceeding to the field to implement the intervention. The following 24 months thus presented a learning curve, as the team (comprised of all co-authors) learned that addressing RMC requires more time, semantic sensitivity and programmatic adaptability than initially envisioned. In this commentary, we describe our breakthroughs and setbacks in the process of promoting RMC within MCSPs work in Tanzania. We do not detail the activities we undertook or the results of these activities. Instead, we begin by highlighting some of the opportunities and challenges that facilitated or interrupted the process of undertaking RMC-focused work and we then discuss our experiences in engaging with partners at national, regional, council, community and inter/intra-agency levels throughout the implementation process. Our hope with this commentary is that by being forthright about our experiences, others may be able to better design and execute similar interventions in similar settings.

## Getting an RMC intervention underway in Tanzania – Opportunities and challenges

From the outset, we learned that unlike other interventions wherein general agreement about the issue, its definition and the steps to address it are straightforward, conversations regarding the mere existence of abuse and the promotion of RMC merit much more conversation and collective agreement (within our own team, and among relevant stakeholders including implementing partners, ministry officials, and authorities at the district, facility and community levels). While the academic literature is rife with research that outlines the existence of abuse – including within the Tanzanian setting – scientific consensus about D & A does not translate to the straightforward development of RMC-promoting interventions, nor does it guarantee political buy-in. Solutions are multi-faceted, and often ignite conversations about social norms as well as personal morality and restraint. This contrasts with conversations about other health interventions, for which the conversation about a health issue or intervention is rather straightforward and relatively impersonal. In our experience, raising the topic of RMC often sparked uneasy reflection among all involved. For example, members within our team as well as partners and ministry officials have oftentimes themselves been subjected to disrespect in personal spheres (e.g. in their families) or professional spheres (e.g. as medical students or service providers), and they may have been socialized during their education to routinely (albeit unwittingly) enact disrespectful behaviors in their daily lives. In this sense, discussing RMC forced us to reflect on our own lives (personal and professional), which could induce discomfort. This process of thoughtful, sometimes painful reflection does not typically occur when implementing programs and designing trainings, but it is nevertheless necessary when confronted with an issue that sits at the intersection of health and human rights.

### Challenges & opportunities – National level partnerships

Perhaps due in part to ill-ease with the topic as described above, our program initially struggled to capture the interest of key partners within the Ministry of Health and Social Welfare.^1^ As an organization, Jhpiego routinely collaborates with ministries in a given country to ensure that programs are enhanced in terms of local ownership and future sustainability. In order to facilitate broad, multi-sector buy-in, Jhpiego convened a high-level stakeholder meeting in July 2015 where researchers, programmers, donors and representatives from relevant local bodies (including the Ministry of Health and Social Welfare, and the Tanzania Chapter of the White Ribbon Alliance) presented on the topic of respectful care [10]. Others who have undertaken research and programming related to RMC in Tanzania and elsewhere gave presentations, as did representatives from the United States Agency for International Development (USAID). Despite this initial, high-level meeting, and the energy and enthusiasm generated during the meeting, our team nevertheless struggled to gain traction with Ministry partners. This could in part be linked to the nature of the topic, but it is also because there is not a specific section of the Ministry that views itself as explicitly tasked with the issue of promoting RMC. Furthermore, similar to ministries in many countries, staff members are overstretched and prefer to focus their energy on existing problems rather than being presented with new ones. The Ministry’s desire to remain focused on existing priorities – and the way in which RMC is inherently complex and multi-tiered – was illuminated during a meeting hosted by the Ministry in which health programming through 2020 was discussed. Jhpiego’s RMC programming representative attended the meeting with the intention to advocate that RMC be included as a key strategy within the 2016 Annual Plan for Reproductive, Maternal, Newborn, Child and Adolescent Health. During breakout sessions of the meeting, various service area groups led by the Ministry discussed texts relevant to their domains (with the ultimate aim of incorporating this text into the annual plan). The RMC representative sought to join a group and advocate for RMC. She first tried to nest herself in a group focused on gender, whereupon she was informed that the gender group is more linked to gender based violence, which is only one sphere of RMC. Requests to join a group focused on clinical care were also rebuffed; with guidance that issues related to dignified care are already addressed through existing capacity building programs including trainings on ethics. The RMC representative persisted, outlining that despite capacity building programs and ethics trainings, research from Tanzania indicated that RMC remained a formidable challenge. Nevertheless the RMC issue was tabled and the annual plan was not modified to further emphasize RMC.

As a team, we began to refer to this type of experience, which was reiterated in later engagements, as the “hot potato phenomenon” wherein key partners and decision makers viewed RMC as a topic they would rather not touch.

### Challenges & opportunities – Regional and district level partnerships

**Fig. 1:**
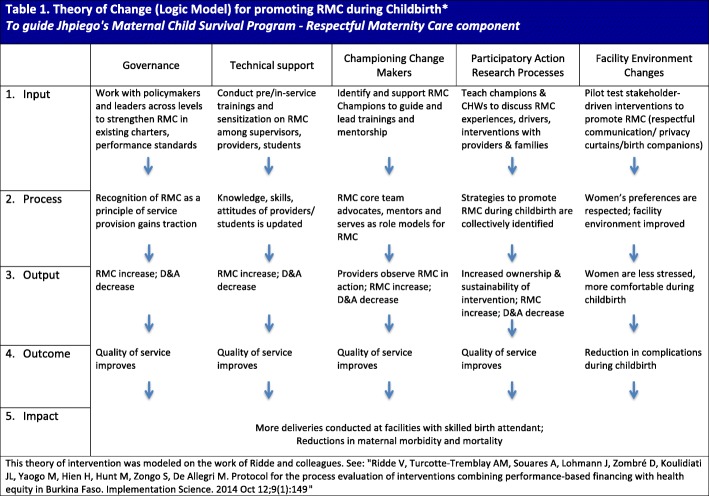
Theory of Change (Logic Model) for promoting RMC during childbirth. *Developed to guide Jhpiego Tanzania’s RMC component in the Maternal and Child Survival Program

While “rigidity” was a term we used among ourselves to describe our efforts to initially engage with Ministry partners, upon taking the issue of RMC to the field we were heartened to see a substantial degree of openness to not only discussing the problem of disrespect but also thinking through solutions. In contrast to our experiences with national stakeholders, stakeholders at regional, district, facility and community levels were proactively reaching out to us to share experiences and seek advice regarding RMC promotion. We suspect this change in tone is linked primarily to the manner in which we, as a team, broached the issue. In the months between attending the Annual Plan meeting and presenting our proposed work to those in districts, our team thought carefully about how to raise the issue of RMC. We also changed our tack slightly. Rather than moving forward with presenting RMC as its own program, we partnered with Jhpiego’s gender team. In doing this, our programmers (who have clinical backgrounds) could harness the skills of programmers trained in social science. We learned from gender-focused colleagues to be careful with terminology. We ultimately avoided employing terms that could spark blame or shame; we did not use the phrase “Disrespect and Abuse” (although it is widely used in the literature) and we framed RMC as a component of quality care. By being open in terms of discussing how disrespect was present in our own lives, we sought to mitigate ‘othering’, or the “process of differentiation and demarcation, by which the line is drawn between ‘us’ and ‘them’ … and through which social distance is established and maintained” [11]. We also modified our usual approach in the sense that we did not come to the district with a codified, ready-made intervention. In line with our learning from engagement with the Ministry, we recognized the need to slow down and gain buy-in before blazing ahead with programs or program refinements. In meetings with regional and district authorities, and other local influential stakeholders (e.g. religious and political leaders), we highlighted that we would like to ultimately develop a multi-pronged intervention (in line with our Theory of Change, see Fig. 1) but first we wanted to gain their thoughts on care during facility delivery, and to also gain insights from relevant voices within communities (especially husband-wife pairs and community leaders), facilities (especially midwives and nurses) and district councils. This led us to ultimately pursue approaches we had not initially considered, namely to partner with district councils and community leaders (such as religious leaders and elected officials) in order to have RMC incorporated into client-provider service charters, which are legal documents enacted at the district level.

### Challenges & opportunities – Intra- and inter-agency partnerships

Thus far, we have emphasized experiences in terms of interacting with governing bodies (the ministry), and district-level actors (district and regional health authorities). Our intra-agency partnering (with Jhpiego’s gender team) was done because we learned that RMC programming is a more complex endeavor than initially envisioned, and because we recognized that the skills, experience and program timeline of the gender work aligned with our own. This partnering was intuitive and fortuitous. Nevertheless, it was not a seamless union. Gender is widely recognized and accepted within the Ministry as an issue that warrants attention. However, the work that is associated with gender centers largely on gender-based violence (GBV). In this respect, we were concerned that by joining RMC with gender, we may limit the scope of understanding since RMC touches on facets of GBV but is also more encompassing. Ultimately, we felt that the common ground between RMC and gender outweighed this difference. Both programs entail discussions regarding power relations, women’s reduced status in society and the normalization of behaviors that are discriminatory toward women. Furthermore, both gender teams and RMC teams have to work across “silos” of health programming rather than being illness specific, and both teams have to engage with actors across a wide spectrum of the health system: community-based sensitizers; religious and political leaders; clinical staff working with facilities at local, district and national levels; health educators within medical, nursing, midwifery and allied health schools; clinical mentors engaged in continuing education and so on. Finally, for both fields to succeed, they must enhance women’s agency and enact shifts in norms (provider norms and cultural norms).

Beyond building partnerships across branches of our organization, we also partnered with institutions in Tanzania that are already engaged in promoting respectful care. Our most direct partnership was forged with the Tanzania Midwives Association (TAMA). TAMA has 25 years of experience working to strengthen and professionalize midwifery across the country. TAMA also has health advocates stationed in the regions and districts where Jhpiego’s maternal health programming is ongoing. We harnessed TAMAs pre-existing network, and together with TAMA managers we implemented RMC and gender-focused workshops for TAMA’s district-level staff. Upon completion of the workshops, participants were named “Gender and RMC Champions”. The Champions then led efforts at district levels to build capacity of facility-based providers on RMC and gender.

We also sought mentorship from other institutions that have undertaken RMC-focused programming. Given the unwieldy nature of RMC, the prospect of creating content seemed daunting (particularly following the impasses encountered at the national level). As we awaited Ministry support for our RMC work, we maintained momentum by drawing upon programming materials and tools from the “Heshima” program in Kenya [12], and the “Staha” [13] and “Uzazi Bora” [10] programs in Tanzania. We used materials from these programs to modify our own work. The programs were slightly different from our own in that they were implementation research projects undertaken to determine what could work to advance RMC; whereas we sought to incorporate these types of approaches into routine programming. We knew of the existence of these programs (and their attendant materials) because delegates from each program presented at the high-level July meeting mentioned above [10], and because all training materials for the Heshima program are freely available online [12]. We cannot overstate the value of engaging with others in a candid, open and collegial manner. By speaking with Staha programmers, we learned of the existence of a Client-Provider Service Charter within Tanzania that had been approved by the Ministry several years earlier and which pointedly describes several facets of respectful care. While the charter is not well known (we could not find an electronic copy of it in our records, online or in the offices of the Ministry of Health), we were able to secure a hard copy in Swahili from a Staha manager. We typed up this Charter, and used it as a foundation to guide conversations with staff at ministry, regional, district and facility levels to underscore the point that respectful care is already viewed as a tenet of quality care in Tanzania and thus merits more concerted attention. When we presented this document to the districts, council lawyers agreed to review it and put it before their respective district councils for a vote – whereby a favorable vote would ensure that the charter becomes a legal document. This is just one example of the manner in which discussing our work with others led us to pursue paths not initially envisioned. Ultimately, we found that the willingness to share information and material saved us time, reduced costs and allowed us to build on what is already known (and agreed upon) rather than unwittingly starting from scratch or “reinventing the wheel”.

Finally, we view the complementary skills of our own team as beneficial to our program. GT and RJM are clinicians, with extensive experience supporting health programming in Tanzania. SC is a midwife who oversees RMC programming globally with Jhpiego. SAM is an Assistant Professor whose dissertation examined RMC in Tanzania. While GT and RJM had not previously worked on RMC programming, they could adapt relevant implementation experience for this issue. SC could compare experiences in Tanzania with those in other settings and reach out to her network of RMC advocates across countries for advice amid setbacks. SAM has minimal experience implementing programs, but could share a wide body of literature on RMC and guide the team in crafting a theory of change and journaling opportunities and setbacks throughout implementation, which formed the foundation for this commentary. While the development of a theory of change was a somewhat painstaking endeavor (see Fig. 1), the team agrees that its creation helped to build consensus within the team about why we were doing what we were doing [14].

## Conclusion

At present, interest within the RMC community is shifting away from more literature on the scope and nature of D & A in favor of studies on how to implement interventions that address RMC. In our experience, the following factors proved most beneficial in supporting our efforts to incorporate RMC into a broader maternal and newborn health program:

- Collecting and referencing a solid body of published literature on the experience of D & A within our context; this blunted conversations about whether D & A existed
- Creating a theory of change among the team; this fostered agreement within the team on how to unpack a complex health problem and kept the team focused throughout implementation
- Recognizing the need to (and being able to) slow the implementation process in the face of rigidity, discomfort and pushback; this allowed the team to gain broader buy-in before moving forward
- Speaking with grassroots-level stakeholders regarding RMC; their enthusiasm sparked our team’s momentum and convinced us that despite challenges we were doing the right thing at the right time
- Partnering with allies within our organization (Jhpiego’s gender team), within the local context (Tanzania Midwives Association) and across other organizations (Heshima, Staha and Uzazi Bora programs); these partnerships allowed us to draw on pre-existing knowledge and to thus prepare for headwinds that others had faced, and to move our program in directions we had not foreseen
- Linking with those who implement gender programming, because the drivers of mistreatment share some of the structural and systemic gender dimensions that affect women’s rights to respectful care

As countries focus efforts on ending preventable maternal and newborn death and achieving the Sustainable Development Goals, improving quality of care is critical. We commend the incorporation of rights-based care in international guidelines related to health programming and practice [15, 16]. In the WHO’s recently published “Standards for Improving Quality of Maternal and Newborn Care in Health Facilities” three of the eight standards listed directly relate to how a woman experiences care and the extent to which she feels she has been treated in a dignified, understandable, respectful and supportive manner [16]. Open and extensive dialogue is now needed to ensure that these standards are operationalized and become the norm. As findings from existing interventions trickle into the published literature, we urge colleagues to also share their program learning experiences -- whether from standalone RMC interventions or from programs like our own that seek to weave components of RMC into existing platforms.

## Footnotes

1 The full name of this ministry is now: Ministry of Health Community Development Gender Elderly and Children
